# Supplementary material for: Mesolimbic dopamine ramps reflect environmental timescales
Source: eLife. 2025 Aug 29;13:RP98666. doi: 10.7554/eLife.98666 (PMC12396814; doi:10.7554/eLife.98666)
Supplement: Supplementary file 1. [file elife-98666-supp1.docx]

**Supplementary File 1 | Statistical details.**

| **Figure** | **Description** | **Test** | **Statistic** | **p value** | **Sample size** |
| --- | --- | --- | --- | --- | --- |
| Figure 1G | Anticipatory lick rate across ITI (long, short) and tone (fixed, dynamic) | Two-way ANOVA | ITI: F(1) = 9.30  Tone: F(1) = 0.30  ITI x Tone: F(1) = 0.029 | ITI: **p = 0.00457  Tone: p = 0.586  ITI x Tone: p = 0.865 | n = 9 mice |
| Figure 1J | Cue onset peak dLight between conditions (LD, SD) | Paired t-test | t(8) = 6.49 | ***p = 1.90x10^-4^ | n = 9 mice |
| Figure 1L | Slope between days (LD condition last day, SD condition first day) | One-sided (LD < SD) paired t-test | t(8) = -2.07 | *p = 0.0363 | n = 9 mice |
| Figure 1L | Slope between days (SD condition last day, SF condition first day) | One-sided (SD > SF) paired t-test | t(8) = 2.35 | *p = 0.0233 | n = 9 mice |
| Figure 1M | Slope across conditions (LF, LD, SD, SF) | One-way ANOVA | F(3) = 8.89 | ***p = 1.97x10^-4^ | n = 9 mice |
| Figure 1M | Slope across conditions (LF, LD, SD, SF) | Tukey HSD test for multiple comparison of means | q = 3.83 | LD vs LF: p = 0.764  LD vs SD: **p = 0.00263  LD vs SF: p = 0.951  LF vs SD: ***p = 1.70x10^-4^  LF vs SF: p = 0.447  SD vs SF: *p = 0.0106 | n = 9 mice |
| Figure 2C | Slope between days (LF condition last day, SD condition first day) | One sided (LF < SD) paired t-test | t(8) = -3.42 | **p = 0.00455 | n = 9 mice |
| Figure 2C | Slope between days (SD condition last day, LD condition first day) | One sided (SD > LD) paired t-test | t(8) = 3.16 | **p = 0.00666 | n = 9 mice |
| Figure 2D | Slope between conditions (LF, SD) | One sided (LF < SD) paired t-test | t(8) = -4.57 | ***p = 9.07x10^-4^ | n = 9 mice |
| Figure 2D | Slope between conditions (SD, LD) | One sided (SD > LD) paired t-test | t(8) = 2.88 | *p = 0.0103 | n = 9 mice |
| Figure 3B | Trial slope regression β given previous ITI (SD condition only) | One-sided (< 0), one sample t-test | t(17) = -3.91 | ***p = 5.58x10^-4^ | n = 18 mice |
| Figure 3C | Trial slope given previous ITI (SD condition only) | Linear regression | t(5343) = -6.19  R^2^ = 0.00711 | ***p = 6.57x10^-10^ | n = 5345 trials |
| Figure 3F | Δ slope regression β given Δ ITI (SD condition only) | One-sided (< 0), one sample t-test | t(17) = -4.17 | ***p = 3.23x10^-4^ | n = 18 mice |
| Figure 3G | Δ slope between Δ ITI <-1 and Δ ITI >1 (SD condition only) | One-sided ((Δ ITI <-1) > (Δ ITI >1)) paired t-test | t(17) = 4.32 | ***p = 2.31x10^-4^ | n = 18 mice |
| Figure 4E | Change in velocity at trial onset (long ITI condition only) | One-sided (> 0), one sample t-test | t(8) = 6.40 | ***p = 1.05x10^-4^ | n = 9 mice |
| Figure 4E | Change in velocity at trial onset (short ITI condition only) | One-sided (> 0) ), one sample t-test | t(8) = 7.93 | ***p = 2.33x10^-5^ | n = 9 mice |
| Figure 4E | Change in velocity at trial onset between conditions (long, short) | Paired t-test | t(8) = 4.25 | **p = 0.00282 | n = 9 mice |
| Figure 4G | Pre-reward velocity between conditions (long, short) | Paired t-test | t(8) = 0.71 | p = 0.497 | n = 9 mice |
| Figure 4I | Cue onset peak dLight between conditions (long, short) | Paired t-test | t(8) = 7.61 | ***p = 6.25x10^-5^ | n = 9 mice |
| Figure 4M | Slope between conditions (long, short) | One-sided (long < short) paired t-test | t(8) = -2.09 | *p = 0.0349 | n = 9 mice |
| Figure 2—figure supplement 1B | Anticipatory lick rate across conditions (LF, SD, LD) | One-way ANOVA | F(2) = 0.26 | p = 0.770 | n = 9 mice |
| Figure 2—figure supplement 1C | Lick rate during ramp window across conditions (LF, LD, SD, SF) | One-way ANOVA | F(3) = 2.24 | p = 0.0929 | n = 18 mice |
| Figure 2—figure supplement 1D | Lick slope during ramp window across ITI (long, short) and tone (fixed, dynamic) | Two-way ANOVA | ITI: F(1) = 1.39  Tone: F(1) = 12.03  ITI x Tone: F(1) = 0.044 | ITI: p = 0.244  Tone: ***p = 9.83x10^-4^  ITI x Tone: p = 0.835 | n = 18 mice |
| Figure 2—figure supplement 4B | Pre-cue dLight slope across conditions (LF, LD, SD, SF) | One-way ANOVA | F(3) = 1.06 | p = 0.375 | n = 18 mice |
| Figure 2—figure supplement 4C | Ramp dLight slope regression β given pre-cue dLight slope (SD condition only) | One-sided (< 0), one sample t-test | t(17) = -1.46 | p = 0.0816 | n = 18 mice |
| Figure 2—figure supplement 4D | Ramp dLight slope given pre-cue dLight slope (SD condition only) | Linear regression | t(5397) = -1.38  R^2^ = 3.53x10^-4^ | p = 0.168 | n = 5399 trials |
| Figure 3—figure supplement 1A | Trial dLight slope regression β given average ITI for previous X trials (SD condition only) | One-sided (< 0 for previous trial, > 0 for previous 2-10 trials) one sample t-test  With Benjamini-Hochberg Procedure | Prev 1: t(17) = -3.91  Prev 2: t(17) = 0.80  Prev 3: t(17) = 1.31  Prev 4: t(17) = 1.48  Prev 5: t(17) = 1.23  Prev 6: t(17) = 1.09  Prev 7: t(17) = 1.32  Prev 8: t(17) = 1.05  Prev 9: t(17) = 1.02  Prev 10: t(17) = 0.76 | Prev 1: **p = 0.00558  Prev 2: p = 0.229  Prev 3: p = 0.202  Prev 4: p = 0.202  Prev 5: p = 0.202  Prev 6: p = 0.202  Prev 7: p = 0.202  Prev 8: p = 0.202  Prev 9: p = 0.202  Prev 10: p = 0.229 | n = 18 mice |
| Figure 3—figure supplement 1B | Trial dLight slope regression β given average ITI for previous X trials (LD condition only) | One-sided (< 0) one sample t-test  With Benjamini-Hochberg Procedure | Prev 1: t(17) = -0.42  Prev 2: t(17) = -3.34  Prev 3: t(17) = -1.94  Prev 4: t(17) = -1.62  Prev 5: t(17) = -1.94  Prev 6: t(17) = -1.61  Prev 7: t(17) = -1.65  Prev 8: t(17) = -1.81  Prev 9: t(17) = -1.31  Prev 10: t(17) = -1.06 | Prev 1: p = 0.339  Prev 2: *p = 0.0194  Prev 3: p = 0.0901  Prev 4: p = 0.0901  Prev 5: p = 0.0901  Prev 6: p = 0.0901  Prev 7: p = 0.0901  Prev 8: p = 0.0901  Prev 9: p = 0.129  Prev 10: p = 0.170 | n = 18 mice |
| Figure 3—figure supplement 2A | dLight slope regression β given dLight onset peak (SD condition only) | One-sided (< 0), one sample t-test  # With Benamini-Hochberg Procedure for all t-tests in Fig 3—fig sup 2 | t(17) = -0.55 | p = 0.538 | n = 18 mice |
| Figure 3—figure supplement 2A | dLight slope given dLight onset peak (SD condition only) | Linear regression  ## With Benamini-Hochberg Procedure for all linear regression in Fig 3—fig sup 2 | t(5397) = -0.53  R^2^ = 5.28x10^-5^ | p = 0.839 | n = 5399 trials |
| Figure 3—figure supplement 2B | dLight slope regression β given dLight onset peak (LD condition only) | One-sided (< 0), one sample t-test  # | t(17) = -0.19 | p = 0.539 | n = 18 mice |
| Figure 3—figure supplement 2B | dLight slope given dLight onset peak (LD condition only) | Linear regression  ## | t(2157) = -0.55  R^2^ = 1.41x10^-4^ | p = 0.839 | n = 2159 trials |
| Figure 3—figure supplement 2C | dLight slope regression β given lick slope (SD condition only) | One-sided (< 0), one sample t-test  # | t(17) = -0.019 | p = 0.985 | n = 18 mice |
| Figure 3—figure supplement 2C | dLight slope given lick slope (SD condition only) | Linear regression  ## | t(5397) = -0.34  R^2^ = 2.13x10^-5^ | p = 0.839 | n = 5399 trials |
| Figure 3—figure supplement 2D | dLight slope regression β given lick slope (LD condition only) | One-sided (< 0), one sample t-test  # | t(17) = -0.88 | p = 0.519 | n = 18 mice |
| Figure 3—figure supplement 2D | dLight slope given lick slope (LD condition only) | Linear regression  ## | t(2157) = -2.53  R^2^ = 2.96x10^-3^ | p = 0.0914 | n = 2159 trials |
| Figure 3—figure supplement 2E | dLight onset peak regression β given lick slope (SD condition only) | One-sided (> 0), one sample t-test  # | t(17) = 1.30 | p = 0.519 | n = 18 mice |
| Figure 3—figure supplement 2E | dLight onset peak given lick slope (SD condition only) | Linear regression  ## | t(5397) = 2.07  R^2^ = 7.96x10^-4^ | p = 0.153 | n = 5399 trials |
| Figure 3—figure supplement 2F | dLight onset peak regression β given lick slope (LD condition only) | One-sided (< 0), one sample t-test  # | t(17) = 0.071 | p = 0.539 | n = 18 mice |
| Figure 3—figure supplement 2F | dLight onset peak given lick slope (LD condition only) | Linear regression  ## | t(2157) = 0.18  R^2^ = 1.43x10^-5^ | p = 0.861 | n = 2159 trials |
| Figure 3—figure supplement 2G | Lick slope regression β given previous ITI (SD condition only) | One-sided (< 0), one sample t-test  # | t(17) = -1.01 | p = 0.519 | n = 18 mice |
| Figure 3—figure supplement 2G | Lick slope given previous ITI (SD condition only) | Linear regression  ## | t(5343) = -1.39  R^2^ = 3.61x10^-4^ | p = 0.440 | n = 5345 trials |
| Figure 3—figure supplement 2H | Lick slope regression β given previous ITI (LD condition only) | One-sided (< 0), one sample t-test  # | t(17) = -0.43 | p = 0.538 | n = 18 mice |
| Figure 3—figure supplement 2H | Lick slope given previous ITI (LD condition only) | Linear regression  ## | t(2103) = -0.40  R^2^ = 7.46x10^-5^ | p = 0.839 | n = 2105 trials |
| Figure 4—figure supplement 1C | Trial duration between conditions (long, short) | Paired t-test | t(8) = 1.02 | p = 0.336 | n = 9 mice |
| Figure 4—figure supplement 1E | Session slope given session IRI (both long & short ITI conditions) | Linear regression | t(52) = -2.61  R^2^ = 0.116 | *p = 0.0118 | n = 54 sessions |
| Figure 4—figure supplement 2B | Trial slope regression β given previous IRI (short ITI condition only) | One-sided (< 0), one sample t-test | t(8) = -0.48 | p = 0.321 | n = 9 mice |
| Figure 4—figure supplement 2C | Trial slope given previous IRI (short ITI condition only) | Linear regression | t(1301) = -2.11  R^2^ = 0.00339 | *p = 0.0355 | n = 1302 trials |
